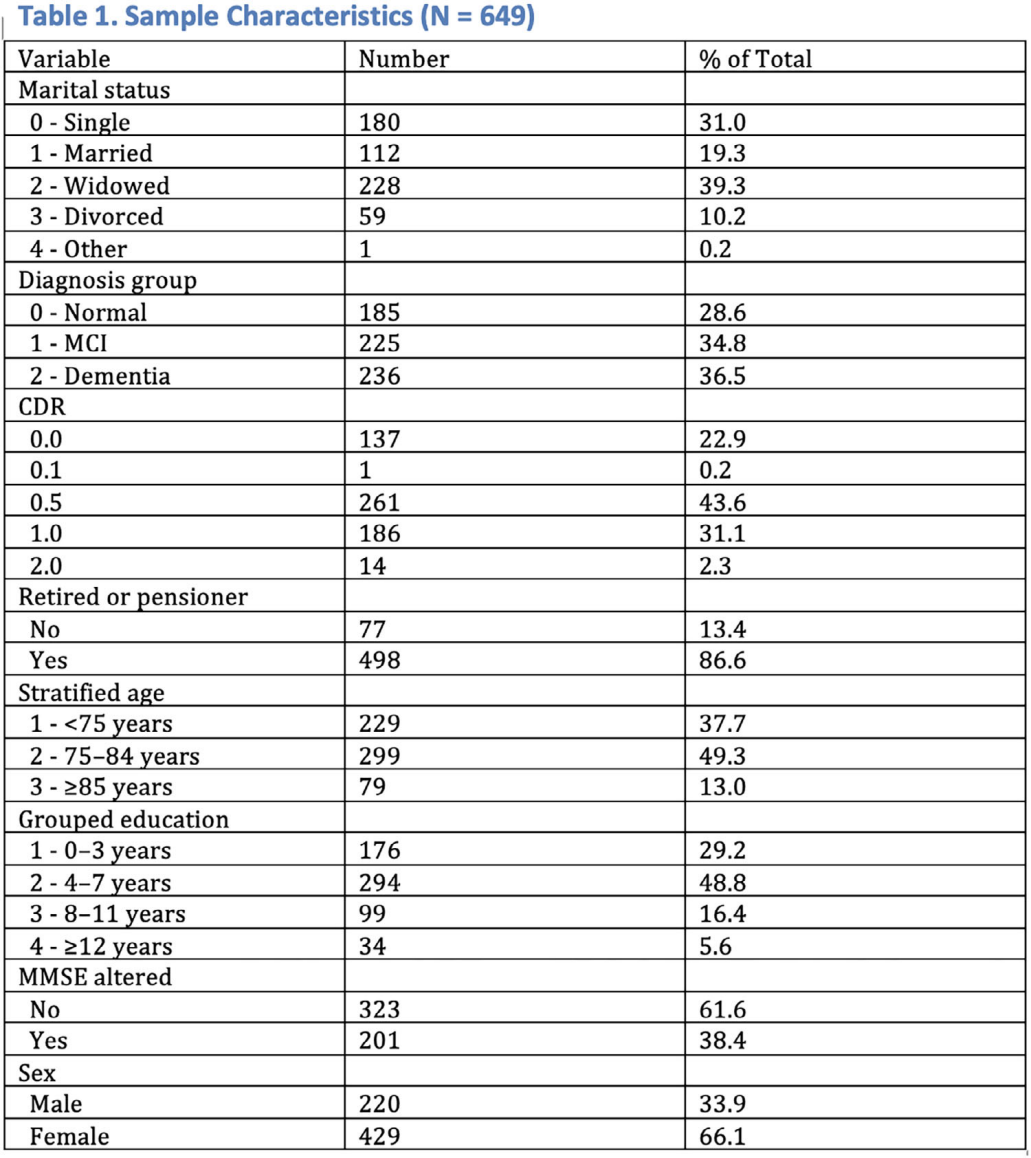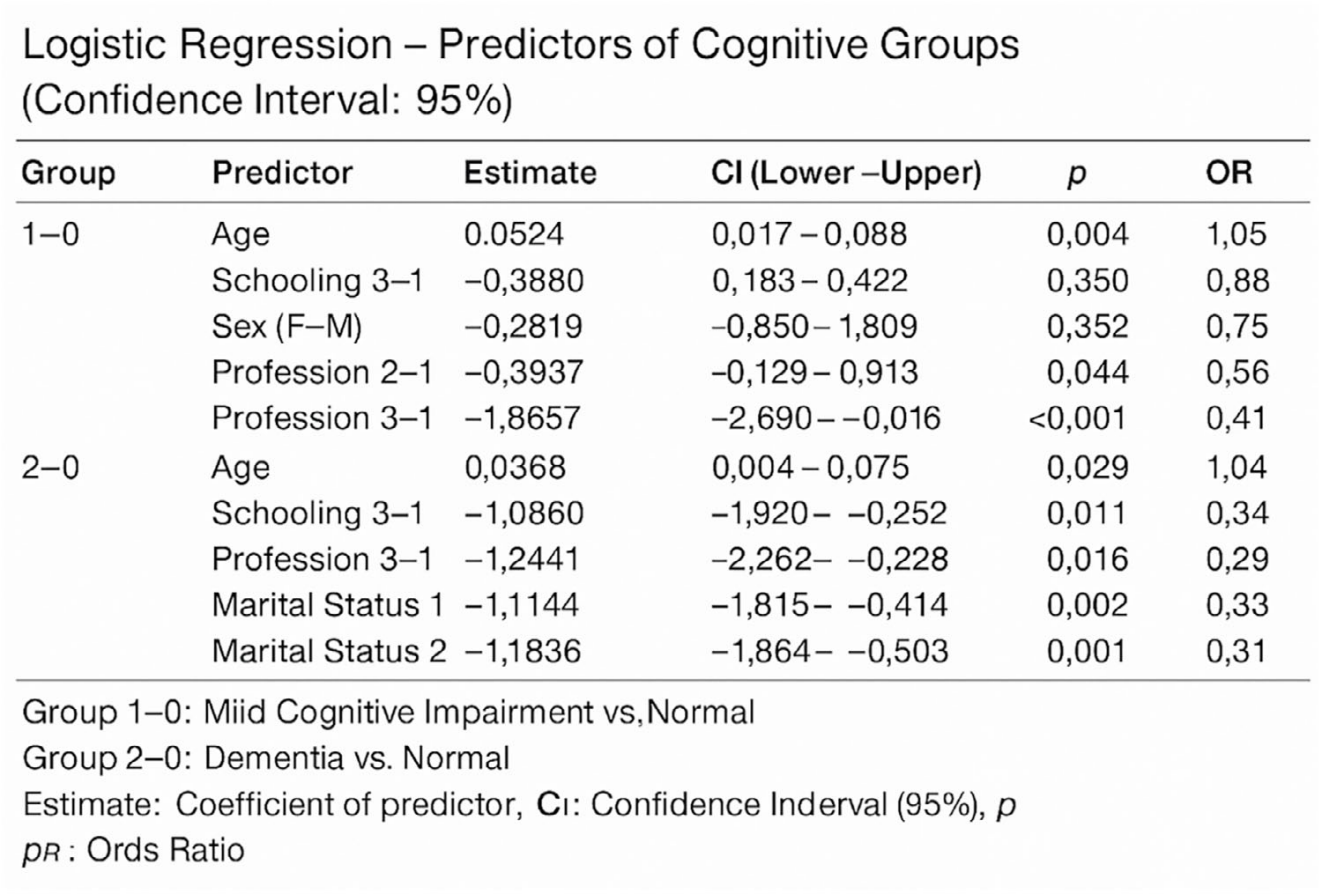# Cognitive profile and Sociodemographic factors in Brazilian Older adults: Insights from a Cross‐Sectional Study

**DOI:** 10.1002/alz70861_108699

**Published:** 2025-12-23

**Authors:** Bárbara Lopes Farace, Joice Coutinho de Alvarenga, Giovanna Correia Pereira Moro, Gabriela Tomé Oliveira Engelmann, Aline Siqueira de Souza, Carolina Andrade Koehne, João Henrique Fonseca, João Marcos Silva Borges, Leonardo Ryuiti Kimoto, Erika de Oliveira Hansen, Julia Kelly Campos, Júlia de Almeida Barreto, Thaise Vallesca Queiroz, Jonas Jardim de Paula, João Vitor da Silva Viana, Julia Cardoso Costa, Marcela Falleiros dos Reis, Rafaela D'Angelo Machado, Marcelle Ferreira Saldanha, Bernardo de Mattos Viana, Maria Aparecida Camargos Bicalho

**Affiliations:** ^1^ Geriatrics and Gerontology Center Clinical Hospital of University of Minas Gerais, Belo Horizonte, Minas Gerais Brazil; ^2^ Cog‐Aging Research Group, Federal university of Minas Gerais, Belo Horizonte, Minas Gerais Brazil; ^3^ Federal University of Minas Gerais, Belo Horizonte, Minas Gerais Brazil; ^4^ Cog‐Aging Research Group, Universidade Federal de Minas Gerais (UFMG), Belo Horizonte, Minas Gerais Brazil; ^5^ Molecular Medicine Postgraduate Program, School of Medicine, Universidade Federal de Minas Gerais (UFMG), Belo Horizonte, Minas Gerais Brazil; ^6^ Sciences Applied to Adult Health Postgraduate Program, School of Medicine, Universidade Federal de Minas Gerais (UFMG), Belo Horizonte, Minas Gerais Brazil; ^7^ Older Adult’s Psychiatry and Psychology Extension Program (PROEPSI), School of Medicine, Universidade Federal de Minas Gerais (UFMG), Belo Horizonte, Minas Gerais Brazil; ^8^ Undergraduate Medicine, Faculty of Medicine, Universidade Federal de Minas Gerais (UFMG), Belo Horizonte, Minas Gerais Brazil; ^9^ Cog‐Aging Research Group, Belo Horizonte, Minas Gerais Brazil; ^10^ Universidade Federal de Minas Gerais, Belo Horizonte Brazil; ^11^ Jenny de Andrade Faria Institute – Outpatient Reference Center for the Elderly, Universidade Federal de Minas Gerais (UFMG), Belo Horizonte, Minas Gerais Brazil; ^12^ Geriatrics and Gerontology Center Clinical Hospital of Universidade Federal de Minas Gerais, Belo Horizonte, Minas Gerais Brazil; ^13^ Undergraduate Medicine, Federal University of Minas Gerais, Belo Horizonte, Minas Gerais Brazil; ^14^ Older Adult’s Psychiatry and Psychology Extension Program I Federal University of Minas Gerais, Belo Horizonte, MG Brazil; ^15^ Neurotec R National Institute of Science and Technology (INCT‐Neurotec R), Faculty of Medicine, Universidade Federal de Minas Gerais (UFMG), Belo Horizonte, Minas Gerais Brazil; ^16^ Universidade Federal de Minas Gerais, Belo Horizonte, Minas Gerais Brazil; ^17^ Department of Psychiatry, School of Medicine, Federal University of Minas Gerais, Belo Horizonte, Minas Gerais Brazil; ^18^ Cog‐Aging Group Research, Brazil, Belo Horizonte, Minas Gerais Brazil

## Abstract

**Background:**

Social aspects play an important role in cognitive performance and may increase the risk of cognitive decline. In Brazil, a country marked by persistent social inequalities, sociodemographic factors may have a more significant impact as risk factors for dementia compared to other countries.

**Method:**

Data from the Cog‐Aging cohort (2012‐2019) were used in a study approved by the UFMG Ethics Committee. Participants were classified into cognitive group based on clinical evaluation, neuropsychological testing, neuroimaging and DSM‐5 criteria. Professional profiles were categorized into low; medium and high intellectual requirements. In the statistical analyses, we used the omnibus ANOVA test and the logistic regression (LR), using a significance level of 5%.

**Result:**

This data included 607 individuals, mean age was 76.8 years (SD 7.1 range 60‐96), 66.1% were female; 77.9% had less than 8 years of formal education; 82.9% were black or *pardo*. The median MMSE score was 23, and 38.4% of individuals had altered MMSE score. The Clinical Dementia Rating (CDR) showed a mean of **0.575** (SD = 0.43). Participants' previous occupations were categorized by intellectual demand. Occupational complexity was significantly associated with cognitive performance (*p* = 0.023). Individuals with medium (*p* = 0.044 — associated with a decreased risk of cognitive impairment) and high (*p* = 0.001 — associated with a substantially reduced risk (OR = 0.189) intellectual demand professions demonstrated a lower likelihood of cognitive impairment compared to those in lower‐demand occupations. The association between sociodemographic factors and cognitive profile were assessed by a linear regression analysis. The model demonstrated a valid relation with an R² of 0.545, indicating that approximately 54.5% of the variance in cognitive status was explained by the independent variables included. The omnibus ANOVA test revealed that CDR (*p* <0,001), occupational complexity (*p* =0,023), sex (*p* =0,010), educational level (*p* =0,007) and age (*p* =0,044) were statistically significant predictors of cognitive status, demonstrating that these variables were associated with degree of cognitive impairment in the sample. On the other hand, retirement and marital status were not significant in the model.

**Conclusion:**

Sociodemographic factors influence cognitive status, emphasizing the need for preventive strategies in vulnerable populations.